# Supplementary figures and images for: Impact of IgG Isotype on the Induction of Antibody-Dependent Cellular Phagocytosis of HIV by Human Milk Leukocytes
Source: Front Immunol. 2022 May 3;13:831767. doi: 10.3389/fimmu.2022.831767 (PMC9110811; doi:10.3389/fimmu.2022.831767)

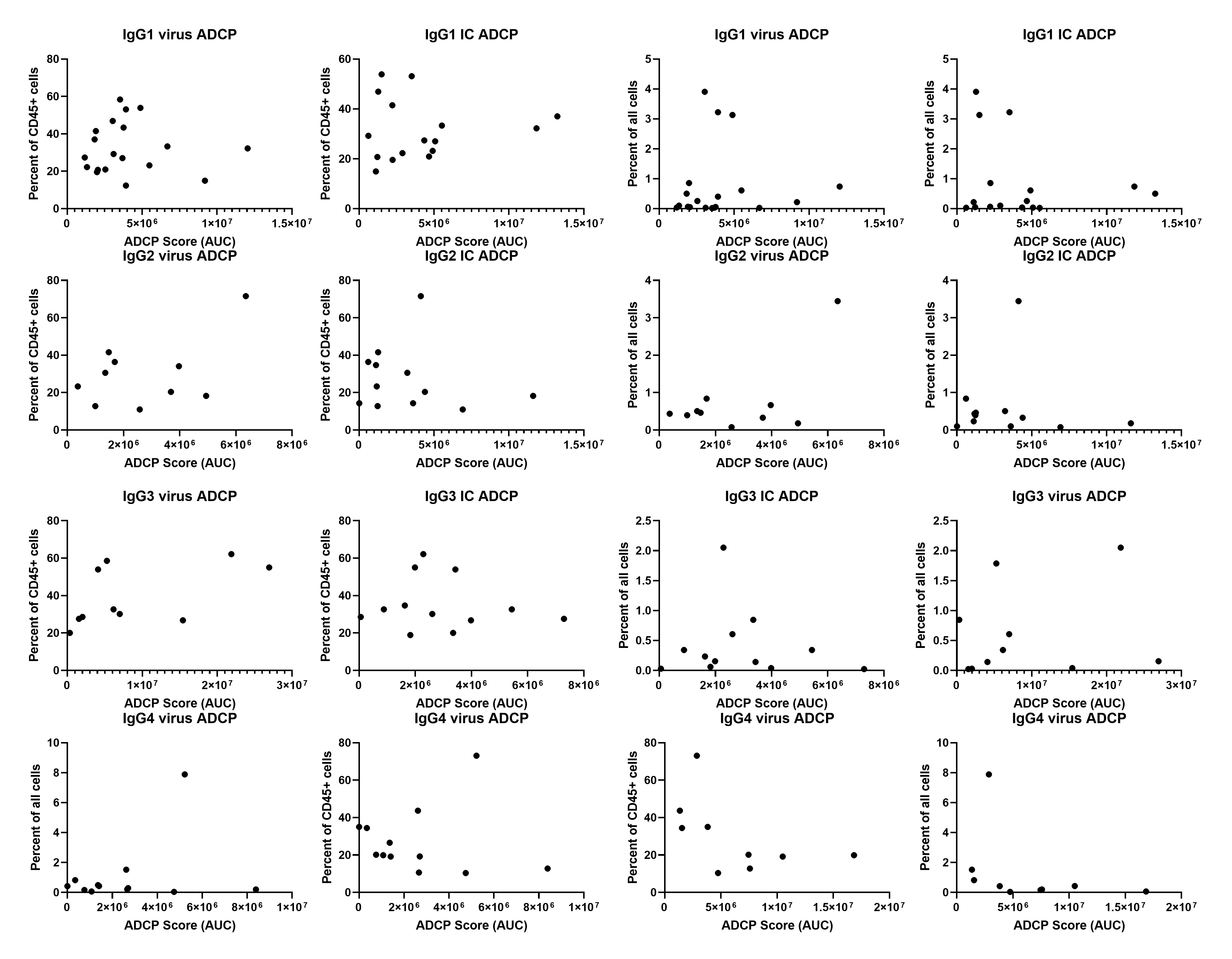

Supplement: Supplementary Figure 1 — Proportion of granulocytes in a milk sample is not related to total ADCP activity of CD45+ leukocytes. Percent granulocytes of total cells and of CD45+ cells for each milk sample analyzed were plotted against the total CD45+ leukocyte ADCP scores for virus ADCP or IC ADCP assays, grouped by IgG isotype. Datasets were assessed for correlation by nonparametric Spearman test. No significant correlations were found. [file Image_1.tif]
